# The interaction of platelet-related factors with tumor cells promotes tumor metastasis

**DOI:** 10.1186/s12967-024-05126-6

**Published:** 2024-04-18

**Authors:** Jie Xue, Jianzhao Deng, Hongwei Qin, Songxia Yan, Zhen Zhao, Lifeng Qin, Jiao Liu, Haiyan Wang

**Affiliations:** 1 Department of Blood Transfusion, The Affiliated Hospital of Qingdao University, 16 Jiangsu Road, Shinan District, Qingdao, 266000 Shandong China; 2 Department of Blood Transfusion, The Central Hospital of Qingdao Jiaozhou, 99 Yunxi River South Road, Qingdao, 266300 Shandong China; 3 Clinical Laboratory, The Central Hospital of Qingdao Jiaozhou, 99 Yunxi River South Road, Qingdao, 266300 Shandong China

**Keywords:** Platelet, Platelet-related factors, Tumor metastasis, Tumor microenvironment, Lung cancer, Breast cancer, Colorectal cancer

## Abstract

Platelets not only participate in thrombosis and hemostasis but also interact with tumor cells and protect them from mechanical damage caused by hemodynamic shear stress and natural killer cell lysis, thereby promoting their colonization and metastasis to distant organs. Platelets can affect the tumor microenvironment via interactions between platelet-related factors and tumor cells. Metastasis is a key event in cancer-related death and is associated with platelet-related factors in lung, breast, and colorectal cancers. Although the factors that promote platelet expression vary slightly in terms of their type and mode of action, they all contribute to the overall process. Recognizing the correlation and mechanisms between these factors is crucial for studying the colonization of distant target organs and developing targeted therapies for these three types of tumors. This paper reviews studies on major platelet-related factors closely associated with metastasis in lung, breast, and colorectal cancers.

## Introduction

Platelets protect tumor cells from mechanical damage caused by natural killer cell lysis and hemodynamic shear stress, which can discharge cytokines, secondary mediators, and growth factors, thus increasing invasion, epithelial–mesenchymal transition (EMT), and extravasation of tumor cells and promoting angiogenesis and vascular remodeling [1–6]. In addition, platelets can induce resistance to anoikis by activating YAP1 signaling [7–9]. Anoikis is a form of apoptosis caused by the loss of extracellular matrix (ECM) adhesion or improper cell adhesion [10]. Anoikis is a barrier to tumor metastasis, and tumor progression and metastasis need to overcome anoikis [11]. Cancer cells can overcome anoikis by changing the integrin repertoire expression [12, 13], promoting EMT [14, 15], and regulating anoikis resistance caused by oxidative stress or hypoxia [16–18]. Cancer cells also act on platelets. Cancer cells can release platelet agonists (ADP, thrombin, and TXA2), induce platelet activation and factor release, and interact with platelets to promote tumor angiogenesis and metastasis [19–24]. Tumor cells from primary tumors invade the matrix and basement membrane, enter the blood flow, and form circulating complexes with leukocytes and platelets [25]. The interplay between platelets and circulating cancer cells produces tumor microemboli, which may block distant organs and promote the interplay of the endothelium [26]. The formation of tumor microemboli is deemed a crucial step in tumor metastasis and colonization during the intravascular phase. Metastasis is a key event in cancer-related deaths [27]. Tumor metastasis is closely associated with platelet-related factors. This strategy opens new prospective avenues for developing therapeutic strategies for lung, breast, and colorectal cancers.

## Lung cancer and platelet-related factors

Lung cancer is the leading cause of cancer-related death worldwide [28]. According to the National Center for Health Statistics, approximately 127,750 individuals in the United States die annually from lung cancer [29]. Non-small cell lung cancer (NSCLC) accounts for approximately 80% of all lung cancers, and its incidence is increasing rapidly [30]. There are usually no obvious in the early stage of lung cancer, so it is necessary to find an appropriate screening method. MicroRNAs (miRNAs), long noncoding RNAs (lncRNAs) and exosomal lncRNAs can be regarded as noninvasive early biomarkers for lung cancer detection [31–33]. At present, many antitumor drugs, such as curcumin, cisplatin and doxorubicin, have been developed for the treatment of lung cancer [34–36]. In addition, treatment methods targeting the AMPK signaling pathway in lung cancer have been proposed [37]. However, it is crucial to clarify the mechanism of lung cancer for its diagnosis and treatment because platelets are related to the growth and metastasis of lung cancer [38].

### Programmed death-ligand 1 (PD-L1)

PD-L1 is the principal ligand of programmed death 1 (PD-1) [39]. PD-1 (CD279) plays an important role in maintaining the tolerance of peripheral and central immune cells by binding to the ligands PD-L1 (B7-H1) and PD-L2 (B7-DC). Platelets from patients with NSCLC express PD-L1, which restrains CD4 + and CD8 + T cells [38, 40, 41]. Blood platelets often contact lung cancer cells both in vivo and in vitro. Platelets can also absorb PD-L1 from cancer cells in a manner dependent on fibronectin, GPIbα, and integrin α5β1 [38]. EGFR activation by EGF stimulation or mutation can decrease the expression of PD-L1 in cancer cells through the p-ERK1/2/p-c-Jun pathway, which plays an important role in the platelet-induced upregulation of PD-L1 in tumor cells [42]. The expression of PD-L1 can predict the response and overall survival (OS) rate in lung cancer patients [43]. PD-L1 is expressed in many malignant tumors and can be transferred from tumor cells to platelets in the form of integrins, thereby playing important roles in tumor immune escape [38, 43]. PD-L1 is related not only to tumor stage and metastasis but also to the prediction of immune checkpoint inhibition (ICI) [38].

### P-selectin

**Fig. 1:**
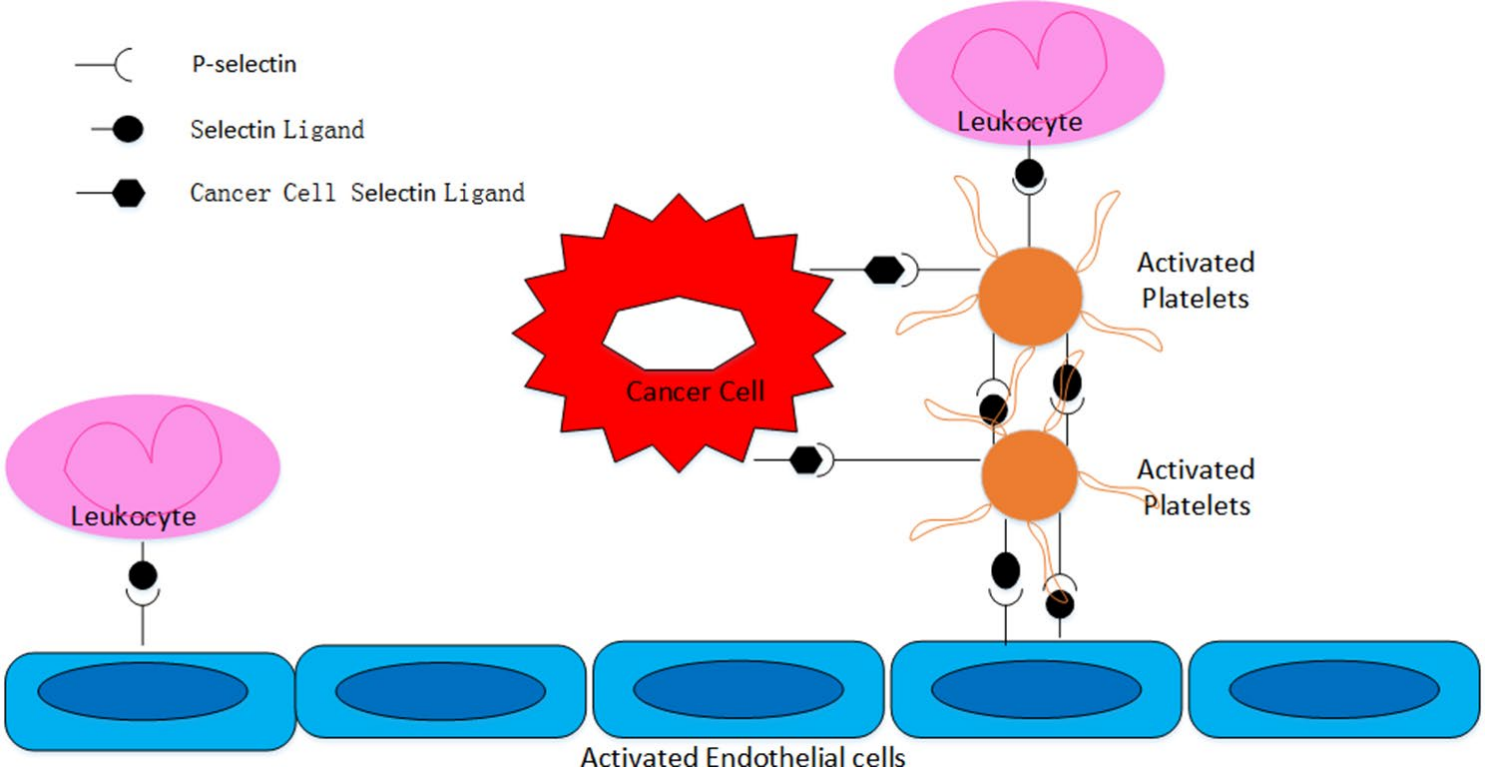
Stimulated endothelial cells and activated platelets express P-selection (CD62P), which interacts with PSGL-1 for leukocyte rolling on stimulated endothelial cells. P-selectin mediates the heterotypic aggregation of activated platelets with cancer cells and adhesion of cancer cells to stimulated endothelial cells. Circulating tumor cells interact with the normal endothelium of the target organ in a leukocyte-like manner and attach to the endothelium of the future metastasis site by using the adhesion molecules of the leukocyte adhesion cascade

Selectin is a key cell adhesion molecule that usually exists in platelet α particles. Generally, no or persistently low expression levels are observed in the resting state. When platelets are stimulated, P-selectin (CD62P) is promptly transferred to their surface through membrane fusion; thus, P-selectin is usually chosen as a marker of platelet activation [44, 45]. Sialyl-LewisX (sLeX) and its isomer sialyl-LewisA (sLeA) are the minimal recognition motifs for all selectins and are synthesized by α2,3-sialyltransferases, α1,3-fucosyltransferases IV or VII, N-acetyl glucosaminyltransferases, and β1,4-galactosyltranferases. The combination of selectins with carbohydrates normally requires a glycoprotein scaffold, and P-selectin glycoprotein ligand-1 (PSGL-1) is the most characteristic ligand assembled at the tips of microvilli on the surface of white blood cells [46–48]. PSGL-1 is expressed on different cell surfaces. PSGL-1 interacts with P-selectin to initiate platelet-mediated cell adhesion. Activated platelets and endothelial cells express P-selectin, which interacts with PSGL-1 to aggregate activated platelets on leukocytes, progressing to activated endothelial cells. P-selectin mediates the aggregation of activated platelets with cancer cells and the adhesion of cancer cells to activated endothelial cells [49]. Circulating tumor cells act on the normal endothelium in a leukocyte-like manner and adhere to the endothelium of the metastatic site via adhesion molecules [50] (Fig. 1). Activated platelets act on lung cancer cells via PSGL-1, leading to distant hematogenous metastasis of lung cancer cells [51].

### Integrins

Integrin is a cell-matrix adhesion molecule that not only provides mechanical engagement of the cell to the extracellular matrix but also transduces signals related to cancer and malignant tumors. Integrins have two primary functions: mechanically linking cells to the extracellular matrix (ECM) and initiating signal transduction pathways. In other words, they serve as both the physical connection between cells and the ECM and as initiators of signaling processes [52]. Integrins are large glycoproteins composed of a group of noncovalently related type I transmembrane α- and β-subunits [53, 54]. There are two integrin subgroups in platelets, β1 and β3, which can compose five human platelet integrins [55]. Two β3 integrins exist on platelets, namely, αIIbβ3 and αvβ3 [56, 57]. Integrins are primary regulators of cell adhesion, diffusion, and migration. Integrins play important roles in promoting oncogenic growth factor receptor (GFR) signaling and GFR-dependent cancer cell invasion, as well as in determining the colonization of metastatic sites and promoting the survival of circulating tumor cells [58].

#### Integrin αIIbβ3

The integrin αIIbβ3 is the main integrin on platelets and is also referred to as the glycoprotein GPIIb/IIIa (CD41/CD61) complex. This integrin is essential for normal platelet function. The integrin αIIbβ3 is also produced in lung cancer cells [59]. Notably, integrin αIIbβ3 can recognize RGD peptide-binding sequences on different adhesive proteins, such as fibrinogen and von Willebrand factor (VWF). The main function of integrin αIIbβ3 is to promote platelet aggregation through its binding with plasma fibrinogen. Its dimeric structure ensures the effective linkage of platelets [60]. Transmitting bidirectional signals is a key feature of integrin αIIbβ3. In the resting state, integrin αIIbβ3 is in an inactive conformation. However, the affinity of the extracellular domain for this ligand is low. Under agonist stimulation, the cytoplasmic tail of integrin αIIbβ3 can bind to intracellular proteins, especially talin or kinin. This combination leads to intracellular and transmembrane separation. The integrin αIIbβ3 complex undergoes a conformational change in its extracellular domain, transitioning from a low-affinity (inactive) state to a high-affinity (active) state for its ligand (fibrinogen) [61]. According to the literature, integrin αIIbβ3 exists in different tumor cells [62–65], promoting cancer cell adhesion and invasion [63–66].

#### Integrin αvβ3

**Fig. 2:**
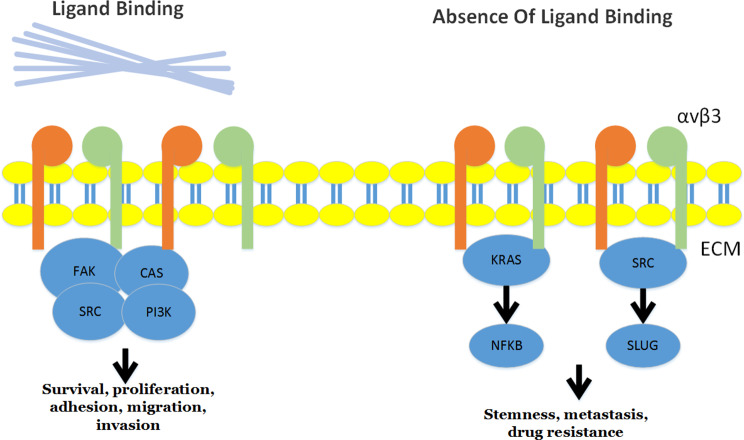
Integrin signaling generated by binding to extracellular matrix (ECM) ligands occurs. In the absence of ligand binding, αvβ3 integrin recruited kras and src to drive cell reprogramming events, which led to phenotypic changes, thus promoting stem dryness, metastasis and drug resistance. Fibrin-fibronection complex induces the activation of αvβ3 integrin, which triggers Survival, prolifera on, adhesion, migra? on, invasion in cancer cells

Integrin αvβ3 is considered a recognized marker of breast, lung, and pancreatic cancers [67, 68]. Integrin αvβ3 can trigger nonanchored cell survival and tumor metastasis without ligand binding [69]. The expression of integrin αvβ3 is necessary for inducing the stem-like properties of lung cancer cells [67]. Integrin αvβ3 is usually not produced by epithelial cells and has been shown to be a remarkable regulator of tumor angiogenesis [70–72]. The fibrin-fibronectin complex induces the activation of integrin αvβ3, which triggers proinvasive EMT signaling and invasive protrusions in cancer cells [73, 74]. In tumor cells, integrin αvβ3 not only phosphorylates the adaptor protein p130 CRK-associated substrate (p130CAS) but also induces adhesion-dependent activation of steroid receptor coactivator (Src) and focal adhesion kinase (FAK). These signaling events lead to the survival, proliferation, and invasion of tumor cells in combination with the ECM [67, 68]. The inhibition of integrin αvβ3 binding to ECM ligands can directionally block endothelial cell-mediated tumor metastasis and angiogenesis. Moreover, integrin-blocking agents have become a potential strategy for targeted therapy [52]. In lung cancers, clusters of integrin αvβ3 emerge on the surface of suspended cells. This clustering is mediated by the interplay between galectin-3 and integrin αvβ3, which is irrelevant to its ligand binding domain [67]. Unligated integrin αvβ3 can drive tumor cells toward a stem-like state, whereas when connected to its ligands, it can contribute to ECM-driven cell invasion and proliferation [68] (Fig. 2). Compared with blocking tumor integrin αvβ3 alone, blocking both platelet integrin αIIbβ3 and tumor integrin αvβ3 simultaneously yields greater antiangiogenic and antitumor effects. These findings indicate that antagonists targeting both platelets and endothelial integrins may have clinical efficacy.

### Autotaxin

Autotaxin (ATX) is a unique member of the nucleotide pyrophosphatase family. ATX induces lysophospholipase D (lysoPLD) activity, which catalyzes lysophosphatidic acid (LPA) production [9]. By producing LPA, ATX and/or lysoPLD can promote tumor progression by providing a favorable microenvironment for tumor cell invasion and angiogenesis [75]. ATX is a multidomain protein consisting of two somatomedin B (SMB1,2)-like domains, a catalytic phosphodiesterase (PDE) domain and a nuclease-like domain [76]. Compared with that in healthy lung tissue, ATX overexpression in lung cancer tissue is significantly related to poorly differentiated or undifferentiated cells [77, 78].

### VEGF and bFGF

Angiogenesis is an extremely important process in the development and metastasis of tumors. Tumor cells attempt to obtain an independent blood supply through a series of processes, including the release of proangiogenic factors and binding to receptors on vascular endothelial cells [79–81]. The interaction between tumor cells and platelets leads to platelet activation. The major angiogenic factors released by the alpha granules of activated platelets include vascular endothelial growth factor (VEGF) and basic fibroblast growth factor (bFGF) [82–84]. Both VEGF and bFGF are considered key regulators of angiogenesis [85, 86]. The VEGF family consists of seven secreted glycoproteins, namely, VEGF-A, -B, -C, -D, -E, -F, and placental growth factor (PlGF). VEGF-A is the most effective factor for angiogenesis [87–89]. In addition, VEGF can be secreted by multiple cell types, including endothelial cells, epithelial cells of the retina, macrophages, stromal cells, and malignant cells. The main receptors of VEGF include VEGFR-1, VEGFR-2, and VEGFR-3 [90, 91]. The binding of VEGF and VEGFR induces receptor dimerization, which leads to the activation of tyrosine kinases in cells, thus exerting their biological effects in cells [87]. In vitro, VEGF and bFGF can induce the proliferation, migration and differentiation of angioblasts [92, 93]. Angioblasts build the primary vascular plexus [94]. However, VEGF and bFGF regulate angiogenesis differentially. Angiogenesis can be driven by VEGF alone but not by bFGF [95]. NSCLC cells can secrete VEGF, increase the number of VEGF and VEGF receptors, and subsequently promote angiogenesis and metastasis [96–98]. However, bFGF- targeted therapy for lung cancer has limitations. Blocking bFGF can inhibit cell growth but promote cell invasion [99]. The overexpression of bFGF may indicate poor prognosis in patients with lung cancer [100].

## Breast cancer and platelet-related factors

Breast cancer (BC) is the most common malignancy in women [101]. Currently, the 5-year overall survival rate of patients with BC without metastasis is > 80% [102]. However, 20–30% of patients with BC develop metastasis after primary tumor treatment [103]. Furthermore, metastasis is the primary cause of death in patients with BC [104], and platelet-related factors are associated with tumor metastasis.

### PD-L1

PD-L1 expression was independently detected in circulating tumor cells and platelets from patients with metastatic BC [105]. The platelet PD-L1 expression level markedly differed between and within patients [105]. This heterogeneity aligns with the varying sensitivities of patients to immune checkpoint inhibition therapy [106, 107].

### P-selectin

P-selectin exists on the surface of endothelial cells and platelets. PSGL-1 is the primary ligand of P-selectin and is responsible for leukocyte rolling on active endothelial cells [108]. In the resting state, P-selectin is expressed at a low level. In the activated state, most P-selectin molecules are transferred from in-granules to platelet membranes [109]. P-selectin initiates interactions between platelets and sialylated fucosylated mucins in circulating tumor cells [25, 110]. Furthermore, P-selectin participates in platelet signaling through protein kinase B (Akt), leading to the phosphorylation of the Src family kinases Fyn and Hck, as well as Erk. These processes appear to be prerequisites for platelet granule secretion and aggregation [111–113]. P-selectin interacts with intracellular talin-1 and subsequently activates integrin GP IIb/IIIa, resulting in the P-selectin-GP IIb/IIIa-talin complex and the accumulation of platelets in tumor tissues [114]. P-selectin can activate additional intracellular signaling pathways that are beneficial for the secretion and aggregation of α-granules and dense granules [109]. Low-molecular-weight heparin (LMWH) combines with P-selectin and simultaneously inhibits the plasma coagulation cascade. Therefore, it is a potentially valuable drug for cancer treatment [109].

### Lysophosphatidic acid

Lysophosphatidic acid (LPA) is a bioactive lipid. It serves as a multifunctional lipid mediator that regulates cell growth, movement, and differentiation [115]. LPA induces several cellular activities, including adenylyl cyclase activation, Ca^2+^ mobilization, and mitogen-activated protein kinase stimulation [116]. There are six distinct G protein-coupled receptors: LPA1, LPA2, LPA3, LPA4, LPA5, and LPA6 [75, 116–118]. LPA is produced by aggregated platelets during tumor cell-induced platelet aggregation. It actss as a paracrine factor in tumor cells through the LPA1 receptor, thereby promoting the proliferation, migration, and secretion of proinflammatory factors [119].

### Autotaxin

ATX can be stored in platelet α-particles. Platelet-derived lysophosphatidylcholine degrades LPA [9]. β3 integrin may bind ATX on the surface of cancer cells/platelets, providing a mechanism for the production of LPA near its receptor, thus enhancing the spread of cancer cells [119]. The interplay between circulating tumor cells and platelets induces platelet aggregation and LPA release. In the blood, LPA acts on tumor LPA1 to promote survival and invasion and may act on platelet LPA5 to promote platelet aggregation [119–121]. Moreover, LPA promotes the migration, invasion, and proliferation of BC cells in vivo [122].

### Integrin αvβ3

Breast and lung cancers share similarities, as both express the integrin αvβ3. Furthermore, their mechanisms of action in cancer cells are similar. Integrin αvβ3 has different functions in tumors, such as promoting angiogenesis, cell proliferation, invasion, and metastasis in different cancers [67, 69]. The integrin αvβ3 ligand L1 cell adhesion molecule (L1-CAM) expressed on BC cells is necessary for BC metastasis to the lungs, where it allows tumor cells to combine and extravasate through the lung endothelium [123]. Specific integrins can dominate the localization and activity of matrix metalloproteases to promote invasive migration. For example, integrin αvβ3 controls matrix metalloproteinase 9 (MMP9) in MDA-MB-435 BC cells [124]. Integrin β3 and KRAS interact via galectin-3 propels to activate RALB. RALB subsequently activates TANK-binding kinase 1, which activates the NF-κB pathway, thus promoting cell survival [69]. Ligated integrins activate FAKs and other downstream signaling molecules, resulting in anchorage-dependent survival and proliferation [125]. However, unligated integrins can induce a form of death called integrin-mediated death (IMD) by activating the apoptosis pathway, thus negatively affecting the malignant characteristics of tumor cells [126].

### VEGF and bFGF

Angiogenesis is an important process related to tumor development [79, 80], which regulated by proangiogenic factors (VEGF, bFGF, and PDGF) and the microenvironment (hypoxia) [127, 128]. In breast cancer cells, VEGFR1 mainly activates the MAPK/ERK1/2 and PI3K/AKT signaling pathways, leading to tumor growth and EMT and thus promoting tumor invasion and metastasis [129]. In addition to promoting angiogenesis, bFGF is involved in plasminogen activator synthesis, cell growth and differentiation, and tumor invasion [130, 131]. Some studies have shown that angiogenesis, tumor growth and metastasis of breast cancer cells can be inhibited by blocking VEGFR1 and VEGFR2 [132–136], and the expression of bFGF is related to a shorter survival time in patients with tumors [137]. Bevacizumab is an effective treatment for metastatic breast cancer targeting VEGF ligands [138]. The success of VEGF- targeted drugs has encouraged the research on targeted therapy for breast cancer, indicating that targeting VEGF is a potentially valuable treatment for breast cancer.

## Colorectal cancer and platelet-related factors

Colorectal cancer (CRC) is a major cause of death worldwide [139]. CRC occurs mainly in the older population, with a median age > 60 years at diagnosis. However, population-based studies have reported that the incidence of CRC is greater in younger populations than in those aged > 50 years [140, 141]. Fecal occult blood tests and colonoscopy are the main screening methods for CRC; however, the invasive nature of colonoscopy limits its application [142]. Therefore, it is important to develop sensitive screening methods for colorectal cancer. Investigations on the expression of platelet-related factors have revealed their association with colorectal cancer cells [142]. These findings could lead to encouraging prospects for future colorectal cancer screening. Understanding the role of platelet-related factors in CRC may offer new insights for the diagnosis, treatment, and prognosis of CRC.

### Platelet-derived growth factor (PDGF)

**Fig. 3:**
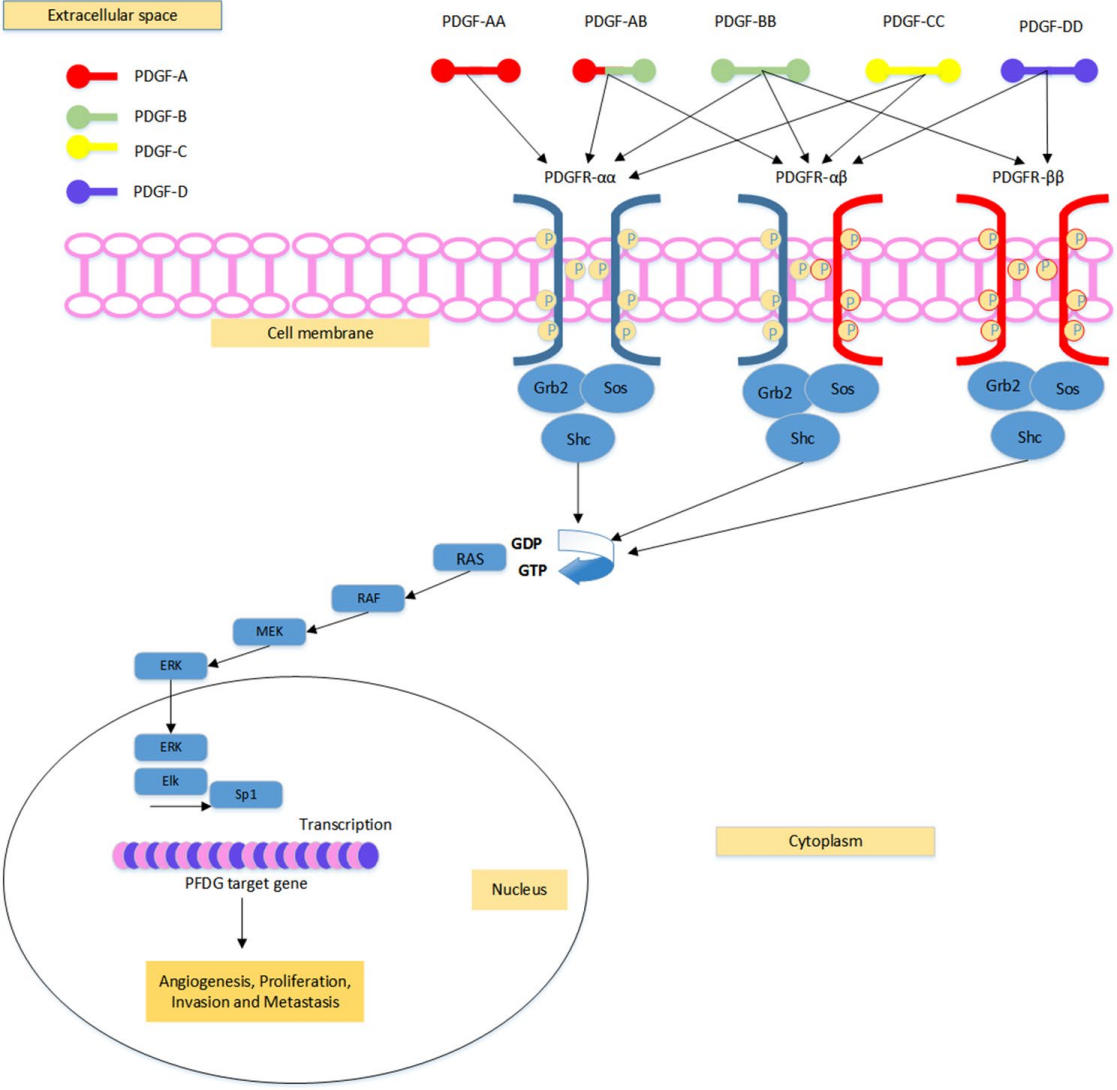
PDGFR-αα binds to all growth factors except PDGF-DD, PDGFR-bb binds to PDGF-BB and PDGF-DD, and PDGFR-αβ binds to all proteins except PDGF-AA. PDGFR/PDGF system initiate a complex cascade of MAP-kinase signaling to activate genes involved in angiogenesis, proliferation, invasion and metastasis

PDGF is a nonhomogeneous molecule that exists in its active form as a dimer through the formation of four chain proteins: PDGF-A, PDGF-B, PDGF-C, and PDGF-D. PDGF-A and PDGF-B can simultaneously form homodimers and heterodimers, whereas the PDGF-C and PDGF-D chains can only form homodimers. PDGF-AB is the most commonly detected form of PDGF-AB in the serum [143]. PDGFs have a series of biological functions and are induced by activating the tyrosine kinase receptors (TKRs) PDGFR-α and PDGFR-β [144]. PDGFR-αα combines with all growth factors, including PDGF-AA, PDGF-AB, PDGF-BB, and PDGF-CC but not PDGF-DD. PDGFR-αβ combines with all growth factors, namely, PDGF-AB, PDGF-BB, PDGF-CC, and PDGF-DD, but not PDGF-AA. PDGFR-β combines with PDGF-BB and PDGF-DD. However, its interaction with PDGF-DD has not been determined [145] (Fig. 3). PDGFR/PDGF signaling results from interactions between dimeric PDGF isoforms and PDGFRs. The specific binding of PDGF ligands to PDGFRs leads to dimerization of these receptors, thus enhancing their stability through interactions with the receptors [146]. Changes in PDGFR family signaling play important roles in CRC. CRC is associated with PDGFR overexpression in tumors and tumor-related stromal cells [145, 147]. Overexpression of PDGFRs in CRC is associated with invasion, angiogenesis, metastasis, low survival rates, and targeted therapies [142]. A high PDGF-AB blood concentration may be an important parameter for CRC recurrence [142]. In summary, PDGF-BB may be involved in the progression of CRC, maintaining angiogenesis by augmenting pericytes within tumors, which is related to disease severity [142]. PDGF-CC has similar mitogenic activity comparable to that of PDGF-AB and PDGF-BB and is also considered to be an important oncogene in the PDGF/PDGFR signaling pathway due to its affinity for both PDGFR-αα and PDGFR-αβ. PDGF-CC induces angiogenesis in vivo [148]. Peripheral blood PDGF-C levels may be useful for the early diagnosis of CRC. In different types of cancers, PDGF-DD is upregulated; however, its role in CRC has not yet been determined [142]. The interplay between platelets and tumor cells improves their survival rate in the blood and promotes tumor metastasis [149, 150].

### Glycoprotein VI (GPVI)

GPVI is a receptor for collagen, laminin, and fibrin [151–153] and can regulate platelet functions such as adhesion, aggregation, and procoagulant activity. GPVI is a member of the Ig superfamily and has two Ig domains (D1 and D2), a stalk containing an O-glycosylation site, and a cytoplasmic tail for binding Src kinase and calmodulin [152, 154]. The signal transduction of GPVI relies on its association with the dimeric Fc receptor chain (FcRγ). GPVI activation results in the phosphorylation of two conserved tyrosine residues that are dependent on Src kinase, which binds to the tandem SH2 domain of Syk. Subsequently, a signaling cascade is initiated, leading to the activation of phospholipase Cγ2 [155]. Glycoprotein VI is thought to bind to galectin-3 (Gal-3) in tumor cells, inducing platelet activation and promoting metastasis in CRC cells [156].

Gal-3 is a member of the β-galactoside-binding lectin family that is located mainly in the cytoplasm [157]. Gal-3 is present in the nucleus and on the cell surface and can be secreted into the circulation [158, 159]. As the main GPVI ligand in tumor cells, Gal-3 induces platelet activation and promotes BC metastasis [160]. The interplay between Gal-3 and GPVI promotes platelet activation, degranulation, and tumor cell transendothelial migration [160].

### Autotaxin

ATX is a unique member of the ectonucleotide pyrophosphatase/phosphodiesterase (ENPP) family, which has lysoPLD activity and can convert lysophosphatidylcholine (LPC) into LPA [161–164]. LPA interacts with G protein-coupled receptors on the cell membrane, which can activate downstream signaling molecules, such as Ras, Rho, PLC, and PI3K [165]. In the early stage of CRC, the expression of ATX is positively correlated with tumor angiogenesis [166]. The up-regulation of ATX is related to cancer invasion and metastasis [9, 167].

### VEGF and bFGF

Related studies have shown that the density of blood vessels at the infiltrating edge of CRC tissue is significantly greater than that in other areas of the tumor [168], and a high density of blood vessels is related to CRC progression and metastasis [169]. Both VEGF and bFGF are considered key regulators of angiogenesis [85, 86]. VEGF-A is the main angiogenic factor in CRC and is related to poor prognosis [170]. VEGFR-1 is expressed in human CRC cells and participates in tumor progression and metastasis [171]. Inhibition of VEGF signaling can lead to a decrease in protein activity related to cell movement, which further reduces the invasion of CRC cells [172]. There is a self-regulating mechanism for angiogenesis in colon cancer. VEGF expression is up-regulated, while bFGF expression is down-regulated [173]. Increased angiogenesis is associated with poor prognosis in patients with CRC, and targeting angiogenesis is a good therapeutic option. In the future, more drugs targeting angiogenesis will be developed, and we need to further explore drugs with high efficacy and minimal adverse effects.

## Conclusions and perspectives

As an important source of circulating angiogenesis-related factors, platelets can affect the tumor microenvironment through interactions with tumor cells. Different platelet-related factors have independent or overlapping effects on the proliferation and metastasis of tumors, and they cross-talk with each other to regulate tumor angiogenesis and vascular integrity. High PD-L1 expression is observed in NSCLC, BC, and CRC [174]. Additionally, P-selectin expression is strongly and positively correlated with PD-L1 expression [38]. In particular, lung and breast cancers highly express P-selectin, integrin αvβ3, VEGF, bFGF, and ATX. Moreover, breast cancer cells express high levels of LPA and ATX in addition to PD-L1, P-selectin, integrin αvβ3, VEGF, bFGF, and PDGF. Integrin αvβ3 promotes bone metastasis through strengthened breast cancer cell adhesion [69]. Colorectal and breast cancers express high levels of ATX, PDGF, VEGF, bFGF, and GPVI. Overexpression of PDGF-AA/BB in patients with stage 4 breast cancer is associated with a relatively shorter survival time [175].

The inhibition of tumor proliferation and metastasis has always been a focus of research. The interaction between platelet-related factors and tumors opens a new direction for research. In lung, breast, and colorectal cancers, we found that tumor cells interact with platelets and that different platelet-related factors have independent or overlapping effects on the proliferation and metastasis of tumors, as these factors can predict the degree of tumor progression, prognosis, and metastasis. Platelet-related factors are interconnected and engage in crosstalk, which introduces a novel concept for tumor treatment. Targeting coexpressed platelet-related factors independently expressed by certain tumor cells to block signaling pathways may inhibit tumor metastasis.

Therapeutic options targeting platelet-related factors are currently being investigated. PD-L1 is expressed in many different types of tumors and platelets in patients with metastatic BC [176]. Immune checkpoint inhibitors targeting PD-L1 and PD-L1 receptors have been verified for tumor treatment [177]. Medi4736 is an antagonist of PD-L1 that can inhibit the growth of human tumors [178]. Low-molecular-weight heparin (LMWH) and unfractionated heparin can bind to P-selectin and inhibit its function [110, 179, 180]. Targeting the activation and inhibition of integrin αIIbβ3 is a promising therapeutic strategy. Adapter protein (ADAP) promotes the activation of integrin αIIbβ3 [181, 182]. Some proteins are also believed to directly bind to the cytoplasmic tails of integrin αIIb or β3 to inhibit the activation of integrin αIIbβ3 [61]. Moreover, α-actin is valuable for keeping integrin αIIbβ3 inactive [183]. Therapeutic drugs targeting integrin αIIbβ3, such as the integrin αIIbβ3 antibody fragment abciximab, antagonists, and small molecule inhibitors, have been used in clinical settings [184]. Therapeutic drugs targeting the integrin αvβ3 molecule, such as cilengitide MRL-123, have been widely investigated for cancer and osteoporosis treatment [185]. Bevacizumab is an antiangiogenic agent, and the FDA approved bevacizumab for the treatment of advanced NSCLC, metastatic breast cancer (mBC) and metastatic colorectal cancer (mCRC) [138, 186, 187]. Inhibitors of Src, Syk, and Tec tyrosine kinases block platelet activation via CLEC-2 and GPVI. Phase II trials using human GPVI-blocking F(ab) ACT-017 have achieved encouraging results [155]. Glenzocimab targets platelet GPVI by binding to the D2 domain of GPVI, inducing steric hindrance and structural modifications, thus inhibiting the interaction between GPVI and its main ligands [188]. PD173074 is an FGFR inhibitor that blocks small cell lung carcinoma (SCLC) growth both in vitro and in vivo [189]. LPA receptors are expressed in the vasculature and brain, which has led to consideration of the toxicity of LPA inhibitors. LPA3 is restricted and abnormally expressed in many cancer lineages, making it a particularly attractive target [75]. Inhibitors against LPA and ATX monoclonal antibodies have been used in clinical trials for treating fibrosis but have not yet entered clinical trials for cancer treatment [190]. GLPG1690 is a new ATX inhibitor [191–193]. PDGFs play important roles in tumor occurrence and are upregulated in many different malignant tumors [194]. At present, numerous drug studies are underway with the aim of inhibiting cancer progression by targeting PDGF. For example, 6B3 is a high-affinity monoclonal antibody that can effectively neutralize PDGF-CC-induced PDGFR-α phosphorylation and activation [195]. MOR8457 is a PDGF antibody that can effectively bind to and neutralize PDGF-BB [196]. Compound P2 can effectively inhibit PDGF-BB-induced autophosphorylation of PDGFR-β with low toxicity [197] (Table 1). However, the interactions of platelet-related factors with tumors are complex and require further exploration. Understanding these new mechanisms and exploring novel approaches to treat tumors in the future are therefore warranted.

**Table 1:** Drug targeting platelet-related factors in the lung, breast and colorectal cancer

Cancer name	Platelet-related factor	Targeted drug	Drug trial phase	Refs
Lung cancer	PD-L1	Medi4736	Clinical Phase	[178]
	P-selection	LMWH	Clinical Phase	[110, 179, 180]
	Integrin αIIbβ3	Abciximab	Clinical Phase	[184]
	Integrin αvβ3	Cilengitide MRL-123	Clinical Phase III	[185]
	ATX	GLPG1690	Clinical Phase	[191, 193]
	VEGF	Bevacizumab	Approval	[186]
	bFGF	PD173074	In vitro and in vivo	[189]
Breast cancer	PD-L1	Medi4736	Clinical Phase	[178]
	P-selection	LMWH	Clinical Phase	[110, 179, 180]
	LPA	LPA monoclonal antibodies	Clinical Phase I	[190]
	ATX	GLPG1690	Clinical Phase	[191, 192]
	Integrin αvβ3	Cilengitide MRL-123	Clinical Phase III	[185]
	VEGF	Bevacizumab	Approval	[138]
	bFGF	PD173074	In vitro and in vivo	[189]
Colorectal cancer	PDGF	6B3, MOR8457, Compound P2	Clinical Phase	[195–197]
	GPVI	Glenzocimab	Clinical Phase	[188]
	ATX	GLPG1690	Clinical Phase	[191]
	VEGF	Bevacizumab	Approval	[187]
	bFGF	PD173074	In vitro and in vivo	[189]

## Data Availability

Not applicable.

## Abbreviations

EMT: epithelial–mesenchymal transition
ECM: extracellular matrix
NSCLC: non-small cell lung cancer
miRNAs: MicroRNAs
lncRNAs: long noncoding RNAs
PD-L1: programmed death-ligand 1
LPA: lysophosphatidic acid
ATX: autotaxin
PDGF: Platelet-derived growth factor
GPVI: Glycoprotein VI
PD-1: programmed death 1
OS: overall survival
ICIs: immune checkpoint inhibitors
sLeX: Sialyl-Lewis X
sLeA: sialyl-Lewis A
PSGL-1: P-selectin glycoprotein ligand-1
GFR: growth factor receptor
vWF: von Willebrand factor
ADAP: Adapter protein
Src: a steroid receptor coactivator
FAK: focal adhesion kinase
lysoPLD: lysophospholipase D
PDE: phosphodiesterase
VEGF: vascular endothelial growth factor
bFGF: basic fibroblast growth factor
PlGF: placental growth factor
BC: breast cancer
Akt: protein kinase B
LMWH: low molecular weight heparin
PDE: phosphodiesterase domain
L1-CAM: L1 cell adhesion molecule
MMP9: matrix metalloproteinase-9
IMD: integrin-mediated death
ITAM: immunoreceptor tyrosine-based activation motif
ENPP: ectonucleotide pyrophosphatase/phosphodiesterase
LPC: lysophosphatidylcholine
Gal-3: Galectin-3
CRC: colorectal cancer
TKRs: tyrosine kinase receptors
mBC: metastatic breast cancer
mCRC: metastatic colorectal cancer
SCLC: small cell lung carcinoma
